# Assessment of Structured Education on Young Surgeons’ Ability to Achieve a Quality Critical View of Safety During Laparoscopic Cholecystectomy: A Pre- and Post-intervention Longitudinal Study

**DOI:** 10.7759/cureus.85303

**Published:** 2025-06-03

**Authors:** Mohamed A El Sayed, Amr A Abd El Naser, Moustafa M Emad, Mohammad A Abd-erRazik

**Affiliations:** 1 Department of Surgery, Defense Industries Medical Center, Cairo, EGY; 2 Department of General Surgery, Ain Shams University, Cairo, EGY

**Keywords:** critical view of safety, customized medical education, laparoscopic cholecystectomy, laparoscopic cholecystectomy complications, post cholecystectomy bile duct injury

## Abstract

Background: Laparoscopic cholecystectomy (LC) is the standard treatment for various gallbladder pathologies. However, bile duct injuries (BDIs) remain a major complication despite advancements in techniques. The critical view of safety (CVS) has been proposed to reduce BDI rates, yet, the “CVS Paradox” suggests that injury rates have remained stable in some centers despite reports of using the CVS technique.

Objective: This study aimed to assess the impact of structured educational sessions on young surgeons’ ability to achieve optimal CVS during LC.

Methods: A pre- and post-intervention longitudinal study was conducted on 40 patients, divided into two groups: Group A (pre-education) and Group B (post-education). Ten junior surgeons performed LCs at two centers, with each performing two procedures before and two after the educational intervention on CVS. Recorded surgical videos were assessed using a 6-point CVS scoring system.

Results: There were no statistically significant differences between the groups in terms of demographic data, preoperative diagnosis, or comorbidities. Intraoperative BDI occurred in 10% of Group A patients, but none in Group B (p=0.147). The CVS was misreported in 50% of Group A cases but in none of Group B (p<0.001). The mean CVS score was significantly higher in Group B (5.45±0.60) compared to Group A (3.80±0.82) (p=0.001). “Satisfactory CVS” achievement was also significantly greater in Group B (95%) versus Group A (40%) (p=0.001).

Conclusion: Structured educational sessions significantly improved the attainment of CVS and the accuracy of its reporting, suggesting that focused training can help reduce complications such as BDI during LC.

## Introduction

Laparoscopic cholecystectomy (LC) is the treatment of choice for various gallbladder pathologies, including acute and chronic cholecystitis, symptomatic cholelithiasis, some cases of biliary dyskinesia, gallstone pancreatitis, and gallbladder masses or polyps [1,2]. While LC is generally considered safe, one of the most serious complications associated with the procedure is bile duct injuries (BDIs) [3], which remains one of the most feared complications following LC due to its association with high morbidity and potential long-term consequences [4].

Efforts to reduce the incidence of BDI during LC have included advancements in equipment, refinement of surgical techniques, and improvements in laparoscopic training. These measures have collectively contributed to a reduction in BDI rates [5-8]. Technological advancements include developing laparoscopic systems equipped with high-resolution cameras, which provide enhanced visualization and improved color differentiation. Other adjuncts like intraoperative cholangiography [9,10] and near-infrared fluorescence imaging with indocyanine green (ICG) dye [11,12] have further improved the ability to delineate biliary anatomy.

The refinement of surgical techniques has been pursued through efforts to standardize the process of identification of key anatomical structures during LC. Techniques such as the critical view of safety (CVS), the fundus-first cholecystectomy, the visualization of the common hepatic duct and common bile duct, and the infundibular approach have been proposed as methods to enhance safety and reduce BDI [13,14]. None of these techniques has been consistently supported by strong clinical evidence. However, the method suggested by Strasberg in 1992, later known as the CVS technique [7], has been recommended by numerous international surgical societies and is considered the standard of care [15].

The use of the CVS technique is associated with lower rates of BDI [6,15-17]. However, paradoxically, centers that have adopted the routine use of the CVS technique do not consistently report lower BDI rates [6,17,18]. This phenomenon has been described as the “CVS Paradox” [19].

One of the causes of the CVS paradox is the failure to achieve a complete CVS consistently. This issue is not attributed to a lack of surgical experience, but rather to inadequate education on the conditions required to achieve a high-quality CVS [20,21]. Consequently, we hypothesize that junior surgeons are more likely to achieve a high-quality CVS during LC after participating in structured educational sessions focused on safe dissection techniques.

## Materials and methods

This pre- and post-intervention longitudinal study was conducted from September 2022 to March 2023. A total of 10 junior surgeons, each with less than eight years of experience in LC, were recruited. The study was conducted at Al Demerdash Hospital (Department of General Surgery, Faculty of Medicine, Ain Shams University) and the Defense Industries Medical Center. The surgeons performed a total of 40 LC procedures, with each surgeon completing four surgeries - two before and two following an educational session focused on the optimal achievement of the CVS.

Inclusion and exclusion criteria

Patients included in the study were those diagnosed with chronic calculous cholecystitis (CCC), gallbladder polyps, or acute calculous cholecystitis. Patients were excluded if they had a history of prior upper abdominal surgeries or required conversion to open cholecystectomy, partial cholecystectomy, or cholecystostomy/drainage. Additionally, patients with difficult cases requiring senior staff intervention or those deemed unfit for surgery were excluded.

Surgical technique

All operations were performed under general anesthesia, with the patient in the reverse Trendelenburg position and the right shoulder elevated. Surgeons were instructed to use either the open technique or the closed technique with a Veress needle at Palmer’s point (left upper quadrant) for initial port insertion. The scope ports were inserted at or near the umbilicus, and other ports were inserted under direct vision. The standard four-port, two-handed technique in the American position was used. Dissection of Calot’s triangle was performed using a combination of blunt dissection (Maryland dissector) and monopolar electrocautery (L-shaped hook). All surgeries were attended and monitored by the principal investigator, with no interventions or comments from the investigator during the procedures. Each surgeon performed four LC cases: two before and two after the educational session, all of which were recorded for later analysis.

Post-operative protocol

Post-operative pain management was provided, and oral fluids were initiated a few hours after surgery. If tolerated, patients were allowed a light breakfast the following morning. Surgical drains, if present, were typically removed on the first post-operative day before discharge. Readmissions due to complications were included in the total post-operative stay. Patients were followed up at the outpatient clinic at one week and four weeks after discharge.

Educational session

The educational sessions were conducted by the investigator and a member of the supervisory team. These sessions included a micro-lecture explaining the three components of the CVS using illustrations and real surgical photos. Additionally, the CVS assessment tool was introduced and discussed with the participants.

Data collection

Surgical videos of each case were recorded, de-identified, and subsequently assessed by senior surgeons who were blinded to both the identity of the operator and whether the surgery was performed before or after the educational session. The videos were evaluated using a 6-point CVS assessment tool, adapted from Sanford and Strasberg [22], to objectively measure the quality of the achieved CVS. The 6-point CVS assessment tool had a minimum score of 0 and a maximum score of 6, with a score of ≥5 considered indicative of a satisfactory CVS.

Ethical considerations

The study received approval from the Research Ethics Committee (REC) of the General Surgery Department at Ain Shams University (IRB 00006379). Informed consent was obtained from all patients after they were provided with a detailed explanation of the procedure and the available alternatives.

Statistical analysis

Recorded data were analyzed using the IBM SPSS Statistics for Windows, Version 23 (Released 2016; IBM Corp., Armonk, New York, United States). Quantitative data with a normal distribution were presented as mean ± standard deviation (SD) and ranges, while non-normally distributed variables were presented as medians with interquartile ranges (IQR). Qualitative variables were reported as frequencies and percentages. Data normality was assessed using the Kolmogorov-Smirnov and Shapiro-Wilk tests.

The following statistical tests were applied: an independent samples t-test for comparing means between two groups when the data were normally distributed. Mann-Whitney U test for two-group comparisons involving non-parametric data. Chi-square test for comparing qualitative data, with Fisher’s exact test used when the expected count in any cell was less than 5.

A 95% confidence interval (CI) was applied, and a margin of error of 5% was accepted. A p-value of <0.05 was considered statistically significant.

## Results

In this study, Group A (n=20) consisted of patients who underwent LC before the educational sessions, while Group B (n=20) comprised patients who underwent LC following the educational intervention. The procedures in both groups were performed by the same cohort of junior surgeons.

There was no statistically significant difference between the two groups with respect to demographic data, including age, gender, and comorbidities (Table 1). Both groups were well-matched in terms of baseline characteristics.

**Table 1:** Demographic data and comorbidities of both groups. ^#^ Independent t-test; ^ Chi-square test BMI: body mass index

	Total (n=40)	Group A (n=20)	Group B (n=20)	Test value	p-value
Age "years"					
Mean±SD	38.15±15.14	36.10±14.34	40.20±16.00	0.728^#^	0.399
Range	16-69	16-65	16-69		
Sex, n (%)					
Female	27 (67.5)	13 (65.0)	14 (70.0)	0.114^	0.736
Male	13 (32.5)	7 (35.0)	6 (30.0)		
BMI					
Mean±SD	30.58±8.51	29.85±8.93	31.30±8.23	0.285^#^	0.597
Range	18-46	18-45	20-46		
Comorbidities, n (%)					
Diabetes Mellitus	5 (12.5)	2 (10.0)	3 (15.0)	0.229^	0.633
Hypertension	6 (15.0)	2 (10.0)	4 (20.0)	0.784^	0.376
Ischemic Heart	1 (2.5)	0 (0.0)	1 (5.0)	1.026^	0.311
Hyperlipidemia	1 (2.5)	1 (5.0)	0 (0.0)	1.026^	0.311

Similarly, no statistically significant difference was observed between the groups regarding pre-operative diagnoses or the necessity for pre-operative endoscopic retrograde cholangiopancreatography (ERCP), as shown in Table 2. This suggests that both groups had comparable pre-operative conditions and clinical profiles. Anatomical variations were encountered in 7.5% of the total patient population during surgery (Table 2), and no significant difference was found between Group A and Group B in this regard.

**Table 2:** Indication of surgery, pre-operative ERCP and anatomical variations in both groups. ^ The chi-square test was used. ERCP: endoscopic retrograde cholangiopancreatography

	Total (n=40)	Group A (n=20)	Group B (n=20)	Test value^	p-value
Diagnosis, n (%)					
Acute Cholecystitis	4 (10.0)	2 (10.0)	2 (10.0)	1.029	0.598
Chronic Cholecystitis	35 (87.5)	17 (85.0)	18 (90.0)		
Polyps	1 (2.5)	1 (5.0)	0 (0.0)		
Pre-operative ERCP, n (%)	0 (0.0)	0 (0.0)	0 (0.0)	0.000	1.000
Anatomical Variations, n (%)					
Double cystic artery	1 (2.5)	1 (5.0)	0 (0.0)	3.027	0.387
Caterpillar anomaly	1 (2.5)	0 (0.0)	1 (5.0)		
Phrygian cap	1 (2.5)	1 (5.0)	0 (0.0)		
Non	37 (92.5)	18 (90.0)	19 (95.0)		

There was no statistically significant difference in operative times between the pre- and post-education groups. Approximately 17.5% of patients underwent additional procedures alongside LC, with the types of surgeries detailed in Table 3. No statistically significant difference was noted between the two groups in terms of the frequency or nature of these additional procedures.

**Table 3:** Comparison of operative time and associated surgeries between the two groups. # Independent t-test; ^ Chi-square test

	Total (n=40)	Group A (n=20)	Group B (n=20)	Test value	p-value
Operative time					
Mean±SD	71.25±26.83	67.50±26.84	75.00±27.35	0.875^#^	0.583
Range	50-300	50-300	50-260		
Associated surgery, n (%)					
Gastric Bypass	1 (2.5)	0 (0.0)	1 (5.0)	2.364^	0.669
Paraumbilical Hernia	3 (7.5)	2 (10.0)	1 (5.0)		
Sleeve Gastrectomy	2 (5.0)	1 (5.0)	1 (5.0)		
Varicocele	1 (2.5)	1 (5.0)	0 (0.0)		
Non	33 (82.5)	16 (80.0)	17 (85.0)		

Post-operative complications were more frequent in Group A, where four patients (20%) experienced complications. These included biliary leakage (n=2), port site hematoma (n=1), and port site infection (n=1). In contrast, Group B had only two patients (10%) with complications, consisting of a port site hematoma (n=1) and a port site infection (n=1) (Table 4). The mean hospital stay for patients in Group A was 1.40 ± 0.99 days, compared to 1.10 ± 0.31 days in Group B. While the length of hospital stay was slightly longer in Group A, this difference was not statistically significant (p > 0.05) (Table 4).

**Table 4:** Post-operative complications and hospital stays. ^#^ Independent t-test; ^ Chi-square test

	Total (n=40)	Group A (n=20)	Group B (n=20)	Test value	p-value
Post-operative complications, n (%)					
No	34 (85.0)	16 (80.0)	18 (90.0)	0.784^	0.376
Yes	6 (15.0)	4 (20.0)	2 (10.0)		
Port Site Hematoma	2 (5.0)	1 (5.0)	1 (5.0)	0.000^	1.000
Biliary Leak	2 (5.0)	2 (10.0)	0 (0.0)	2.105^	0.147
Port Site Infection	2 (5.0)	1 (5.0)	1 (5.0)	0.000^	1.000
Hospital stay "days"					
Mean±SD	1.25±0.74	1.40±0.99	1.10±0.31	1.66^#^	0.205
Range	1-5	1-5	1-2		

The CVS was reported as achieved in 18 patients (90%) in Group B, compared to 17 patients (85%) in Group A. Despite a slightly higher report of attainment rate in Group B, the difference between the two groups was not statistically significant.

There is a marked difference in the frequency of misreporting of CVS between the groups. In Group A, in 10 patients (50%), there was misreporting of attaining CVS, whereas no misreporting was documented in Group B. This difference was statistically significant (p < 0.001) (Table 5).

**Table 5:** Reporting, misreporting, and assessment score for the critical view of safety (CVS). ^#^ Independent t-test; ^ Chi-square test and Fisher’s exact test; * Significant difference IQR: interquartile range

	Total (n=40)	Group A (n=20)	Group B (n=20)	Test value	p-value
Reported CVS attainment, n (%)					
No	5 (12.5)	3 (15.0)	2 (10.0)	0.229^	0.633
Yes	35 (87.5)	17 (85.0)	18 (90.0)		
Misreported cases, n (%)					
No	30 (75.0)	10 (50.0)	20 (100.0)	6.382^	0.001*
Yes	10 (25.0)	10 (50.0)	0 (0.0)		
Assessment score for CVS					
Mean±SD	4.43±1.26	3.80±0.82	5.45±0.60	6.858^#^	0.001*
Median (IQR)	5 (3-6)	4 (3-4)	5 (5-6)		
Range	2-6	2-5	4-6		
Level of score for CVS, n (%)					
Satisfactory Critical View of Safety	27 (67.5)	8 (40.0)	19 (95.0)	9.552^	0.001*
Unsatisfactory Critical View of Safety	13 (32.5)	12 (60.0)	1 (5.0)		

A highly statistically significant difference was observed in the median score for CVS assessment between the groups. The median score in Group B was 5 (range: 2-5), compared to 4 (range: 4-6) in Group A, with a p-value of 0.001. This indicates a significant improvement in the quality of CVS attainment post-education (Table 5).

Furthermore, the rate of satisfactory achievement of CVS was significantly higher in Group B, with 19 patients (95%) reaching satisfactory CVS, compared to only eight patients (40%) in Group A. This difference was also highly statistically significant, with a p-value of 0.001 (Table 5).

## Discussion

Since its introduction in 1985, LC quickly gained popularity over open and mini-cholecystectomy [23,24] due to its minimally invasive nature and faster recovery times. However, this increase in adoption was associated with higher rates of BDIs compared to open cholecystectomy, prompting researchers to standardize techniques aimed at reducing such injuries [15,25]. Despite the efforts, none of the techniques have been evaluated through randomized controlled trials (RCTs), due to the impractical sample sizes such trials would require [19].

The technique describing the CVS was first reported in 1992 by Strasberg et al. [7], however, the term “Critical View of Safety” was coined in 1995 [7]. The CVS comprises three key elements: (1) separating the lower third of the gallbladder from the liver bed; (2) clearing the hepatocystic triangle of any fat or connective tissue; and (3) ensuring that only two structures, the cystic duct and the cystic artery, remain attached to the gallbladder.

The CVS technique was widely accepted as a method to standardize the identification of key structures during LC, aiming to reduce BDI. Some evidence has shown that adopting this technique is associated with lower BDI rates [15,16]. Yet, paradoxically, BDI rates have remained relatively stable even in centers that have implemented the CVS technique [17], as mentioned earlier, referred to as the “CVS Paradox” [19].

Surprisingly, even after decades of its introduction, some surgeons may still confuse CVS with the infundibular technique, which is associated with higher rates of BDI. While surgeons frequently report in their operative details that the CVS was achieved, the actual adherence to proper technique can sometimes be uncertain, which may contribute to persistent complication rates [6].

This study aimed to evaluate the impact of educational interventions on young surgeons' ability to achieve an optimal CVS during LC. Forty patients were included in this study and divided into two groups: Group A (n=20) included surgeries performed before the educational sessions, and Group B (n=20) consisted of surgeries performed after the educational sessions.

The study population had a mean age of 38.15 years, with 67.5% being female. No statistically significant differences in age or sex were observed between the groups. This finding aligns with previous studies reporting a predominance of middle-aged females [11]. Gallstone disease is more common in women, especially during their fertile years, with females being twice as likely to form stones compared to men [26].

The most common diagnosis was CCC, present in 87.5% of patients, with no cases requiring pre-operative ERCP. There were no statistically significant differences between the two groups regarding pre-operative diagnosis (p=0.598) and the need for ERCP (p=1.000). These findings differ from much of the existing literature, which often reports a higher incidence of acute cholecystitis or the need for pre-operative interventions like ERCP [14,24,27]. This discrepancy may be attributed to exclusion bias, where patients anticipated to have more difficult LC procedures are typically assigned to senior surgeons. As a result, the study population may have been shifted toward less complex cases. The introduction of the educational session did not lead to a reduction in the duration of the procedures.

In our study, intraoperative BDI occurred in 10.0% of patients in Group A. One case (5%) was identified and managed intraoperatively by a senior surgeon, while the other was diagnosed post-operatively and treated accordingly. No BDI cases were observed in Group B. The difference was not statistically significant (p=0.147). This finding aligns with some studies, including that of Avgerinos et al. [16].

Reviewing photographic or video recordings of LC and comparing them with surgeons' written operative reports may reveal discrepancies in reported CVS attainment. The visual evidence provided by the photographs or videos can sometimes refute the accuracy of the written records, thereby identifying instances of potential misreporting regarding the achievement of CVS [28]. Surgeons self-reported achieving CVS in 85.0% of Group A and 90.0% of Group B, with no significant difference between the groups. However, reviewing procedure recordings revealed that CVS was misreported in 50.0% of Group A but in none of Group B, with a highly significant difference (p=0.001). This suggests substantial improvement in proper reporting after the educational intervention.

The mean CVS assessment score was 3.80±0.82 in Group A and 5.45±0.60 in Group B, showing a highly significant difference (p=0.001). Also, “Satisfactory CVS” achievement was significantly higher in Group B (95.0%) compared to Group A (40.0%) (p=0.001). These findings are consistent with Stefanidis et al., who observed improved CVS scores following training [29], and other studies, which reported significant score increases post-training, indicating that surgical education plays a key role in improving safety outcomes during LC [22,30].

Limitations

This study has several limitations. First, the sample size was relatively small, comprising only 40 patients and 10 junior surgeons across two centers. This limits the generalizability of the findings. Second, the study did not include senior surgeons, who might present different learning curves or experiences in achieving CVS. Third, the study lacked a control group, relying solely on pre- and post-intervention comparisons within the same cohort. Finally, potential exclusion bias may exist, as more complex cases were likely assigned to senior surgeons, thus under-representing difficult cases in the study population.

## Conclusions

This study demonstrates that structured educational sessions focused on achieving the CVS during LC significantly improve junior surgeons' ability to safely perform the procedure. Post-education, there was a marked increase in satisfactory CVS attainment, highlighting the importance of targeted training. Further research with larger sample sizes, randomized trials, and long-term follow-up is necessary to confirm the broader applicability and impact of such educational interventions.
